# LncRNA ADAMTS9-AS2 inhibits gastric cancer (GC) development and sensitizes chemoresistant GC cells to cisplatin by regulating miR-223-3p/NLRP3 axis

**DOI:** 10.18632/aging.103314

**Published:** 2020-06-09

**Authors:** Niansheng Ren, Tao Jiang, Chengbo Wang, Shilin Xie, Yanwei Xing, Daxun Piao, Tiemin Zhang, Yuekun Zhu

**Affiliations:** 1 Department of Colorectal Surgery, The First Affiliated Hospital of Harbin Medical University, Harbin 150001, Heilongjiang, China

**Keywords:** gastric cancer, pyroptotic cell death, lncRNA ADAMTS9-AS2, miR-223-3p, NLRP3 inflammasome

## Abstract

The role of LncRNA ADAMTS9-AS2 in the regulation of chemoresistance of gastric cancer (GC) is largely unknown. Here we found that LncRNA ADAMTS9-AS2 was low-expressed in GC tissues and cells compared to their normal counterparts. In addition, LncRNA ADAMTS9-AS2 inhibited miR-223-3p expressions in GC cells by acting as competing endogenous RNA, and the levels of LncRNA ADAMTS9-AS2 and miR-223-3p showed negative correlations in GC tissues. Of note, overexpression of LncRNA ADAMTS9-AS2 inhibited GC cell viability and motility by sponging miR-223-3p. In addition, the levels of LncRNA ADAMTS9-AS2 were lower, and miR-223-3p was higher in cisplatin-resistant GC (CR-GC) cells than their parental cisplatin-sensitive GC (CS-GC) cells. LncRNA ADAMTS9-AS2 overexpression enhanced the cytotoxic effects of cisplatin on CR-GC cells, which were reversed by overexpressing miR-223-3p. Furthermore, LncRNA ADAMTS9-AS2 increased NLRP3 expressions by targeting miR-223-3p, and upregulation of LncRNA ADAMTS9-AS2 triggered pyroptotic cell death in cisplatin treated CR-GC cells by activating NLRP3 inflammasome through downregulating miR-223-3p. Finally, the promoting effects of LncRNA ADAMTS9-AS2 overexpression on CR-GC cell death were abrogated by pyroptosis inhibitor Necrosulfonamide (NSA). Collectively, LncRNA ADAMTS9-AS2 acted as a tumor suppressor and enhanced cisplatin sensitivity in GC cells by activating NLRP3 mediated pyroptotic cell death through sponging miR-223-3p.

## INTRODUCTION

Gastric cancer (GC) is a common malignancy in the digestive system [1, 2], although the number of inpatient admissions for GC have decreased over the past decades, the healthcare burden and cost related to it has increased significantly [3]. Currently, surgical resection [4], radiotherapy [5], chemotherapy [6] and combined therapy [7] remain the primary therapies for GC treatment in clinic. Cisplatin is the first-line chemotherapeutic drug for GC treatment [8, 9], and cisplatin induces GC cell death by triggering DNA damage response (DDR) [10, 11]. However, long-term cisplatin treatment stimulates cancer stem cells (CSCs) to differentiate into heterogeneous GC cells [12–14], as a result, chemoresistant GC cells appears and seriously limits the therapeutic efficacy of cisplatin for GC treatment [12–14]. Additionally, cell pyroptosis is a type of programmed cell death characterized by NLRP3 inflammasome activation and inflammatory cytokines secretion [15, 16]. Recent study found that cisplatin induced pyroptotic cell death in lung cancer cells by targeting Caspase-3/Gasdermin E (GSDME) signaling cascade [17], nevertheless, it is still unclear whether cisplatin induced GC cell pyroptosis.

Long non-coding RNAs (LncRNAs) involved in the regulation of gastric cancer pathogenesis according to their differential biological functions [18, 19], for example, LncRNA MEG3 served as a tumor suppressor [19], while LncRNA HOXA11-AS acted as an oncogene [18] in GC development. Aside from GC progression, LncRNAs regulated chemoresistance of GC cells to chemotherapeutic drugs, such as oxaliplatin [20], gemcitabine [21] and cisplatin [22, 23]. Specifically, LncRNA HOTAIR promoted cisplatin resistance in GC cells [23], but LncRNA CRAL had the opposite effects and reversed cisplatin resistance in chemoresistant GC cells [22]. LncRNA ADAMTS9-AS2 located in chr3:64684720-64809891, which had recently been identified as a tumor suppressor in GC progression [24], and upregulation of LncRNA ADAMTS9-AS2 inhibited GC development by activating PI3K/Akt pathway [24], however, the detailed mechanisms are still unclear. In addition, LncRNA ADAMTS9-AS2 decreased chemoresistance in clear renal cell carcinoma [25], increased temozolomide resistance in glioblastoma [26] and enhanced tamoxifen resistance in breast cancer [27], but no publications reported LncRNA ADAMTS9-AS2 regulated cisplatin resistance in GC cells.

MicroRNAs (miRNAs) are a class of small non-coding RNA molecules that regulated cell functions by targeting the 3’ untranslated regions (3’UTRs) of target genes [28, 29]. Recent studies reported that miRNAs regulated GC pathogenesis and resistance to chemotherapeutic drugs including cisplatin [30, 31]. MiR-223-3p played an oncogenic role in multiple cancers [32–34], and miR-223-3p overexpression promoted GC cell proliferation and invasion [34]. Aside from that, miR-223-3p involved in the regulation of cisplatin-resistance of pancreatic cancer [35], osteosarcoma [36] and GC [37], especially, miR-223-3p promoted cisplatin resistance of GC cells via targeting F-box and WD repeat domain containing 7 (FBXW7) [37]. In addition, overexpression of miR-223-3p alleviated cell pyroptosis in age-related macular degeneration [38] and endothelial cells [39] by inactivating NLRP3 inflammasome, which suggested that miR-223-3p was capable of modulating cell pyroptosis. Of note, researchers identified that miR-223-3p was the downstream target and could be negatively regulated by LncRNA ADAMTS9-AS2 in lung cancer [40].

Based on the above literatures, we aimed to investigate whether LncRNA ADAMTS9-AS2 regulated GC pathogenesis and cisplatin induced GC cell pyroptosis, and uncover the potential molecular mechanisms. This study will give some insights into the role of LncRNA ADAMTS9-AS2/miR-223-3p/NLRP3 pathway in the regulation of GC progression and chemoresistance to cisplatin.

## RESULTS

### LncRNA ADAMTS9-AS2 and miR-223-3p were aberrantly expressed in GC tissues and cells

**Figure 1:**
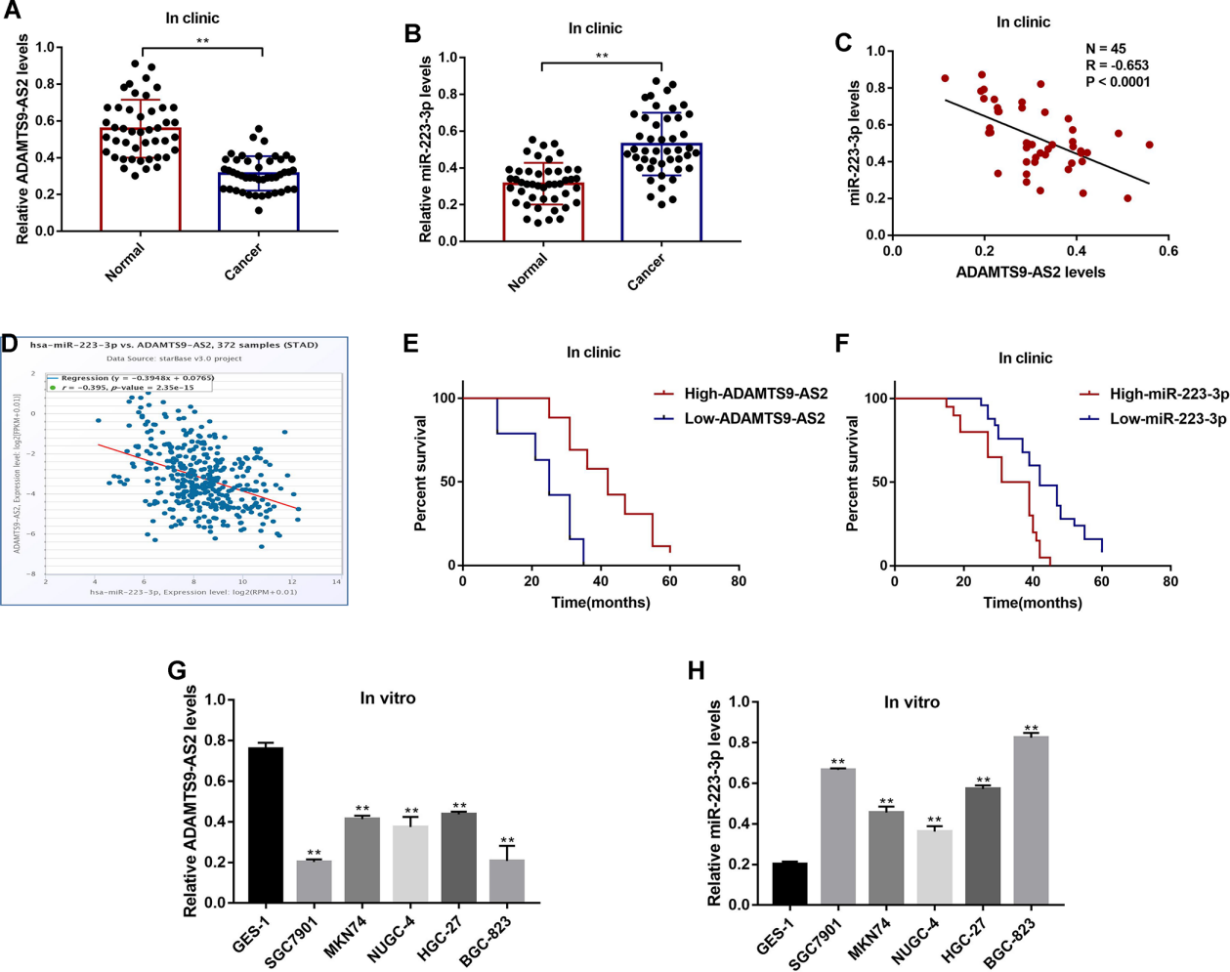
**The expression status of LncRNA ADAMTS9-AS2 and miR-223-3p in GC clinical specimens and cell lines.** Real-Time qPCR was used to examine the levels of (**A**) LncRNA ADAMTS9-AS2 and (**B**) miR-223-3p in cancer tissues and adjacent normal tissues collected from GC patients. (**C**) Pearson correlation analysis was conducted to analyze the correlation of LncRNA ADAMTS9-AS2 and miR-223-3p in GC tissues. (**D**) Pan-cancer analysis was performed to analyze the correlation of LncRNA ADAMTS9-AS2 and miR-223-3p for 372 specimens from the patients with stomach adenocarcinoma (STAD). (**E**, **F**) Kaplan-Meier survival analysis was performed to determine prognosis of GC patients with differential LncRNA ADAMTS9-AS2 and miR-223-3p expressions. Real-Time qPCR was used to measure the levels of (**G**) LncRNA ADAMTS9-AS2 and (**H**) miR-223-3p in GES-1 cells and GC cells. Each experiment repeated at least 3 times. **P < 0.01.

The GC tissues and their paired normal adjacent tissues (N = 45) were collected, and Real-Time qPCR was conducted to determine the expressions of LncRNA ADAMTS9-AS2 and miR-223-3p in the tissues. The results showed that LncRNA ADAMTS9-AS2 was low-expressed (Figure 1A), while miR-223-3p was high-expressed (Figure 1B) in GC tissues compared to their corresponding normal tissues. The expression levels of LncRNA ADAMTS9-AS2 and miR-223-3p were negatively correlated in GC tissues (Figure 1C), which were validated by the online Pan-cancer correlation analysis (http://hopper.si.edu/wiki/mmti/Starbase) for 372 specimens from the patients with stomach adenocarcinoma (STAD) (Figure 1D). In addition, LncRNA ADAMTS9-AS2 was low-expressed, and miR-223-3p was high-expressed in GC tissues from the patients with tumor size (> 3), lymphatic invasion (yes) and TNM stage (III/IV), but did not correlate with other clinical parameters, such as patient age and gender (Table 1). Furthermore, the Kaplan-Meier survival analysis suggested that patients with low-expressed LncRNA ADAMTS9-AS2 (Figure 1E) and high-expressed miR-223-3p (Figure 1F) had a worse prognosis and shorter survival time. Next, we investigated the above results *in vitro* by using the human gastric epithelial cell line GES-1 and GC cell lines (SGC7901, MKN74, NUGC-4, HGC-27 and BGC-823), which also showed negative correlations (Figure 1G, 1H). The results showed that the levels of LncRNA ADAMTS9-AS2 were lower (Figure 1G), but miR-223-3p were higher (Figure 1H) in GC cells comparing to the GES-1 cells.

**Table 1:** The clinicopathological characteristics of GC patients.

**Features**	**Cases**	**LncRNA ADAMTS9**		***P* value**	**miR-223-3p**		***P* value**
		**High**	**Low**		**High**	**Low**	
Age (year)				0.532			0.873
≥ 50	20	11	9		8	12	
<50	25	15	10		12	13	
Gender				0.631			0.521
Male	15	6	9		5	10	
Female	30	20	10		15	15	
Tumor size (mm)				0.004			0.019
≤ 3	19	13	6		14	5	
> 3	26	13	13		6	20	
Lymphatic invasion				0.043			0.031
Yes	12	5	7		8	4	
No	33	21	12		12	21	
TNM stage				0.011			0.045
I/II	21	11	10		9	12	
III/IV	24	15	9		11	13	

### LncRNA ADAMTS9-AS2 regulated GC cell functions by sponging miR-223-3p

**Figure 2:**
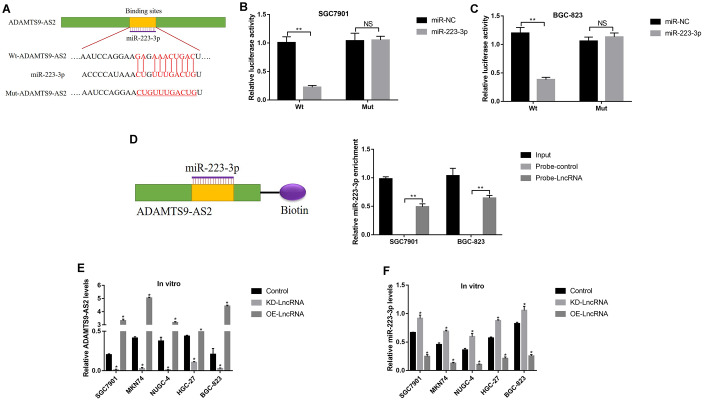
**LncRNA ADAMTS9-AS2 acted as a RNA sponge to regulate miR-223-3p in GC cells.** (**A**) The targeting sites of LncRNA ADAMTS9-AS2 and miR-223-3p were predicted by using the online starBase software (http://hopper.si.edu/wiki/mmti/Starbase). Dual-luciferase reporter gene system was used to verify the binding sites in (**B**) SGC7901 cells and (**C**) BGC-823 cells, respectively. (**D**) RIP was performed to measure the binding abilities of LncRNA ADAMTS9-AS2 and miR-223-3p. Real-Time qPCR was used to examine the expression levels of (**E**) LncRNA ADAMTS9-AS2 and (**F**) miR-223-3p in GC cells. Each experiment repeated at least 3 times. “NS” represented “no statistical significance”, *P < 0.05, **P < 0.01.

**Figure 3:**
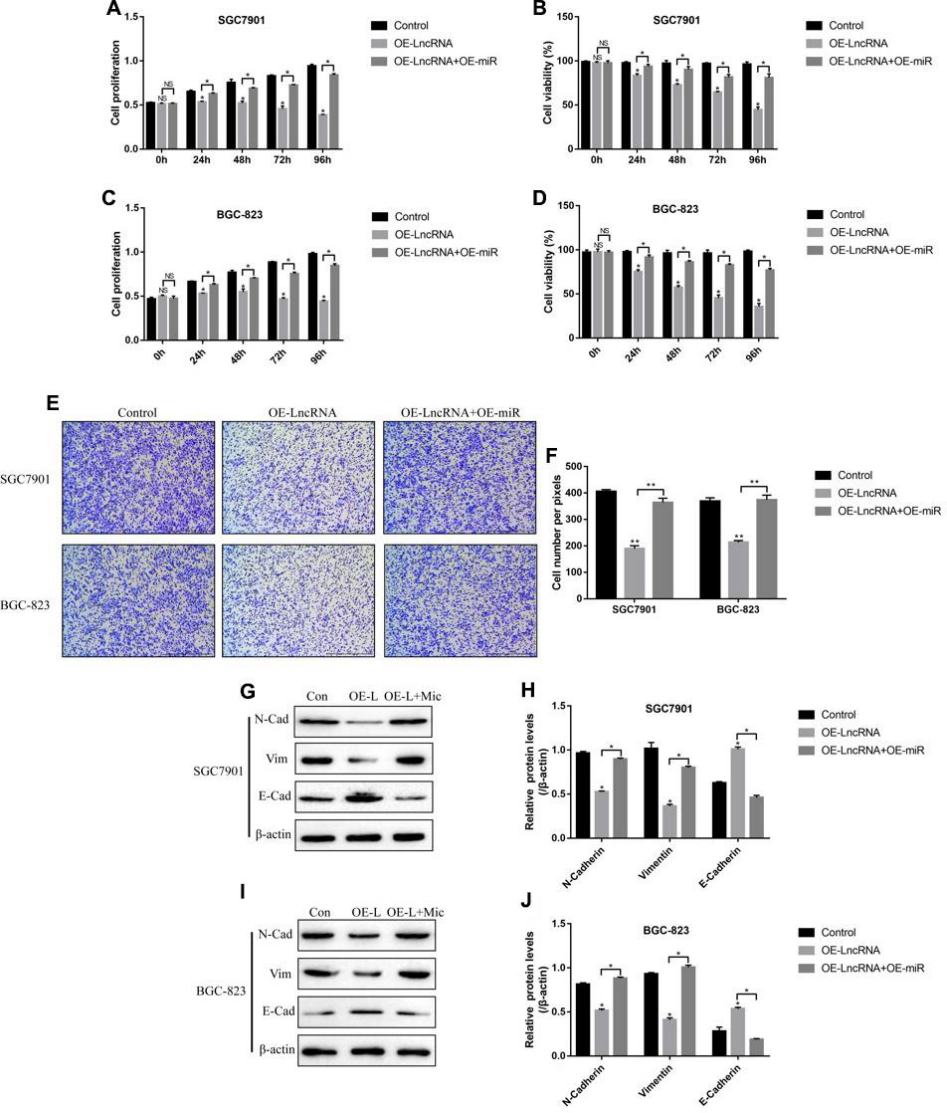
**LncRNA ADAMTS9-AS2 regulated GC cell proliferation, viability, mobility and EMT by targeting miR-223-3p.** CCK-8 assay was used to measure cell proliferation in (**A**) SGC7901 cells and (**C**) BGC-823 cells. Cell counting assay by trypan blue staining method was performed to determine cell viability in (**B**) SGC7901 cells and (**D**) BGC-823 cells. (**E, F**) Transwell assay was conducted to measure cell migration in GC cells. Western Blot was employed to examine the expressions of EMT associated proteins (N-cadherin, E-cadherin and Vimentin) in (**G**, **H**) SGC7901 cells and (**I, J**) BGC-823 cells. Each experiment repeated at least 3 times. “NS” represented “no statistical significance”, *P < 0.05, **P < 0.01.

Previous studies reported that LncRNA ADAMTS9-AS2 acted as a RNA sponge for miR-223-3p [40], which was also validated in this study. The targeting sites of LncRNA ADAMTS9-AS2 and miR-223-3p were predicted by using the online starBase software (http://hopper.si.edu/wiki/mmti/Starbase) (Figure 2A), and validated by the dual-luciferase reporter gene system (Figure 2B, 2C). Specifically, the wild-type (Wt) and mutant vectors (Mut) for LncRNA ADAMTS9-AS2 were co-transfected with miR-223-3p mimic into GC cells (SGC7901 and BGC-823). The results showed that miR-223-3p overexpression significantly inhibited luciferase activity in cells transfected with Wt-LncRNA ADAMTS9-AS2 instead of Mut-LncRNA ADAMTS9-AS2 (Figure 2B, 2C). Consistently, the above results were validated by the LncRNA ADAMTS9-AS2 probe pull-down assay (Figure 2D). In addition, the vectors were successfully delivered into GC cells to overexpress and knock-down LncRNA ADAMTS9-AS2 (Figure 2E), respectively. The results showed that overexpression of LncRNA ADAMTS9-AS2 decreased the levels of miR-223-3p in GC cells (Figure 2F). As expected, downregulated LncRNA ADAMTS9-AS2 had opposite effects on miR-223-3p levels (Figure 2F). Previous publications found that LncRNA ADAMTS9-AS2 inhibited lung cancer progression by targeting miR-223-3p [40], hence we investigated whether LncRNA ADAMTS9-AS2/miR-223-3p axis regulated GC development in a similar manner. The CCK-8 assay and cell-counting assay results showed that LncRNA ADAMTS9-AS2 overexpression inhibited GC cell proliferation (Figure 3A, 3C) and viability (Figure 3B, 3D), which were reversed by transfecting cells with miR-223-3p mimic (Figure 3A–3D). Similarly, the transwell assay results showed that LncRNA ADAMTS9-AS2 inhibited GC cell migration by targeting miR-223-3p (Figure 3E, 3F). Furthermore, the epithelial-mesenchymal transition (EMT) markers (N-cadherin, E-cadherin and Vimentin) were also determined and the results showed that overexpressed LncRNA ADAMTS9-AS2 inhibited N-cadherin and Vimentin, while promoted E-cadherin expressions in GC cells, which were all reversed by overexpressing miR-223-3p in GC cells (Figure 3G–3J).

### Differential expression status of LncRNA ADAMTS9-AS2 and miR-223-3p in CS-GC and CR-GC cells

**Figure 4:**
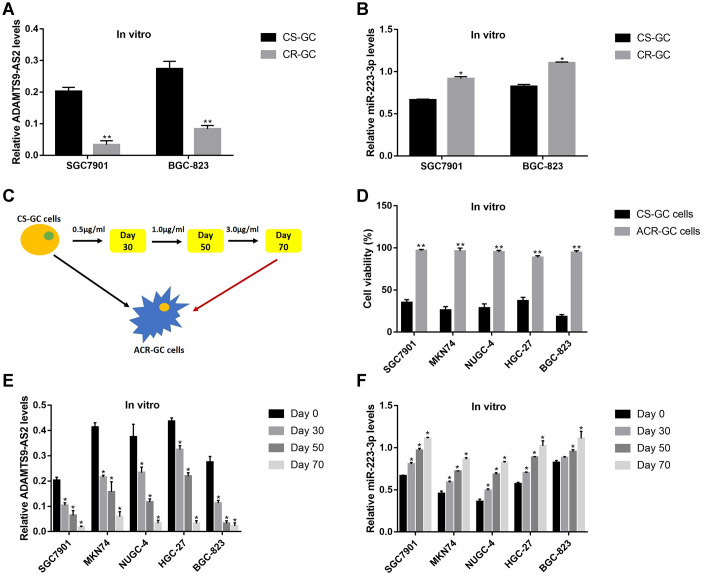
**The expression patterns of LncRNA ADAMTS9-AS2 and miR-223-3p were changed by long-term cisplatin stimulation in GC cells.** Real-Time qPCR was used to examine the expression levels of (**A**) LncRNA ADAMTS9-AS2 and (**B**) miR-223-3p in CS-GC and CR-GC cells. (**C**) The schematic diagram for the production of ACR-GC cells involved in this study. (**D**) Trypan blue staining assay was performed to evaluate cell viability in CS-GC cells and ACR-GC cells. Real-Time qPCR was conducted to examine the expression levels of (**E**) LncRNA ADAMTS9-AS2 and (**F**) miR-223-3p in GC cells treated with continuous low-dose cisplatin stimulation. Each experiment repeated at least 3 times. “NS” represented “no statistical significance”, *P < 0.05, **P < 0.01.

Since both LncRNA ADAMTS9-AS2 and miR-223-3p participated in the regulation of resistance of cancer cells to chemotherapeutic drugs [25, 37], further experiments were conducted to explore the role of LncRNA ADAMTS9-AS2/miR-223-3p axis in the modulation of cisplatin-resistance in GC cells. The Real-Time qPCR results showed that the levels of LncRNA ADAMTS9-AS2 were lower (Figure 4A), while miR-223-3p were higher (Figure 4B) in cisplatin-resistant GC (CR-GC) cells (SGC7901/DDP and BGC-823/DDP) comparing to their corresponding parental cisplatin-sensitive GC (CS-GC) cells (SGC7901 and BGC-823). To validate the above results, the CS-GC cells (SGC7901, MKN74, NUGC-4, HGC-27 and BGC-823) were treated with continuous low-dose cisplatin in a stepwise manner to generate acquired cisplatin-resistant GC (ACR-GC) cells (Figure 4C) according to the previous study [41]. Further results validated that we had successfully inducted ACR-GC cells, which were resistant to high-dose cisplatin stimulation (Figure 4D). In addition, long-term low-dose cisplatin stimulation decreased LncRNA ADAMTS9-AS2 levels (Figure 4E), while increased miR-223-3p levels in GC cells (Figure 4F). The above results suggested that continuous low-dose cisplatin stimulation altered the expression patterns of LncRNA ADAMTS9-AS2 and miR-223-3p in GC cells, which might render GC cells chemoresistance to high-dose cisplatin.

### LncRNA ADAMTS9-AS2/miR-223-3p axis regulated the cytotoxic effects of cisplatin on CR-GC cells by inducing pyroptotic cell death

**Figure 5:**
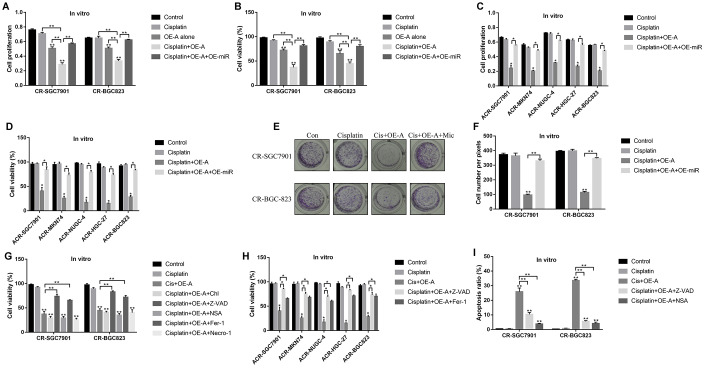
**LncRNA ADAMTS9-AS2 regulated chemoresistance of GC cells to cisplatin by modulating cell pyroptosis.** CCK-8 assay was performed to determine cell proliferation abilities in (**A**) CR-GC cells and (**C**) ACR-GC cells. Trypan blue staining assay was conducted to measure cell viability in (**B**) CR-GC cells and (**D**) ACR-GC cells. (**E**, **F**) Colony formation assay was used to detect colony formation abilities of CR-SGC7901 cells and CR-BGC-823 cells. Cell viability in (**G**) CR-GC cells and (**H**) ACR-GC cells were detected by trypan blue staining assay. (**I**) FCM assay (Related to Supplementary Figure 1) were performed to determine cell apoptosis ratio in CR-GC cells. (“OE-A” represented “overexpressed LncRNA ADAMTS9-AS2”, “OE-miR” represented “overexpressed miR-223-3p”, “Mic” represented “miR-223-3p mimic”). Each experiment repeated at least 3 times. “NS” represented “no statistical significance”, *P < 0.05, **P < 0.01.

Since LncRNA ADAMTS9-AS2/miR-223-3p axis was closely associated with cell functions [40] and aberrantly expressed in CR-GC cells, we next investigated whether targeting this axis rescued CR-GC cells’ sensitivity to cisplatin by manipulating their expressions in CR-GC cells. The results showed that high-dose cisplatin had little effects on both CR-GC cells and ACR-GC cells (Figure 5A–5D), and upregulation of LncRNA ADAMTS9-AS2 inhibited cell proliferation and viability in CR-SGC7901 and CR-BGC-823 cells to a very limited extent (Figure 5A, 5B). However, LncRNA ADAMTS9-AS2 overexpression enhanced the cytotoxic effects of high-dose cisplatin on CR-GC cells and ACR-GC cells, which were abrogated by upregulating miR-223-3p (Figure 5A–5D). Consistently, overexpressed LncRNA ADAMTS9-AS2 inhibited colony formation abilities of high-dose cisplatin treated CR-GC cells, which were reversed by miR-223-3p overexpression (Figure 5E, 5F). Previous studies reported that cisplatin inhibited cancer progression by triggering autophagic cell death [42], apoptosis [43], pyroptosis [17], ferroptosis [44] and necroptosis [45]. Therefore, further experiments were conducted to investigate the underlying mechanisms of LncRNA ADAMTS9-AS2 overexpression induced cell death in cisplatin treated CR-GC cells. To achieve this, the CR-GC and ACR-GC cells were pre-treated with inhibitors for autophagy (chloroquine), apoptosis (Z-VAD-FMK), pyroptosis (NSA), ferroptosis (ferrostatin-1) and necroptosis (necrostatin-1). The results showed that only inhibitors for apoptosis and pyroptosis, instead of autophagy, ferroptosis and necroptosis, abrogated the detrimental effects of overexpressed LncRNA ADAMTS9-AS2 on CR-GC cells treated with high-dose cisplatin (Figure 5G, 5H). Further FCM (Figure 5I, Supplementary Figure 1) assay results validated that LncRNA ADAMTS9-AS2 overexpression increased apoptosis ratio of CR-GC cells treated with high-dose cisplatin, which were reversed by Z-VAD-FMK and NSA.

### LncRNA ADAMTS9 activated NLRP3 inflammasome by downregulating miR-223-3p

**Figure 6:**
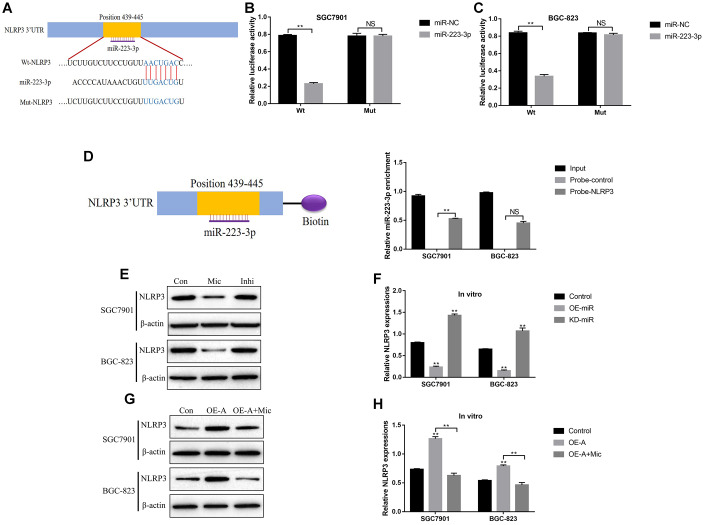
**LncRNA ADAMTS9-AS2 regulated NLRP3 inflammasome in GC cells by sponging miR-223-3p.** (**A**) The targeting sites of miR-223-3p and NLRP3 mRNA were predicted by the online starBase software (http://hopper.si.edu/wiki/mmti/Starbase). Dual-luciferase reporter gene system was employed to validate the binding sites of miR-223-3p and NLRP3 mRNA in (**B**) SGC7901 cells and (**C**) BGC-823 cells. (**D**) RIP assay was used to evaluate the binding ability of miR-223-3p and 3’ UTR regions of NLRP3 mRNA. (**E**–**H**) Western Blot was performed to detect the expression levels of NLRP3 in SGC7901 and BGC-823 cells. (“Mic” represented “miR-223-3p mimic” and “Inhi” represented “miR-223-3p inhibitor”). Each experiment repeated at least 3 times. “NS” represented “no statistical significance”, *P < 0.05, **P < 0.01.

Activation of NLRP3 inflammasome was a crucial procedure in pyroptotic cell death [15, 46], and previous publications found that miR-223-3p inhibited cell pyroptosis by targeting NLRP3 inflammasome [47–49]. Hence it was reasonable to hypothesize that LncRNA ADAMTS9 might regulate NLRP3 inflammasome in GC cells by targeting miR-223-3p. The binding sites of miR-223-3p and 3’ UTR regions of NLRP3 were predicted by the online starBase software (http://hopper.si.edu/wiki/mmti/Starbase) (Figure 6A). Further dual-luciferase reporter gene system assay validated that miR-223-3p significantly inhibited luciferase activity in GC cells (SGC7901 and BGC-823) co-transfected with wild-type NLRP3 (Wt-NLRP3) instead of mutant NLRP3 (Mut-NLRP3) vectors (Figure 6B, 6C). Consistently, the RNA pull-down assay results verified the direct association between miR-223-3p and 3’UTR of NLRP3 mRNA (Figure 6D). In addition, the expression levels of NLRP3 in GC cells were decreased by overexpressing miR-223-3p, and increased by knocking down miR-223-3p (Figure 6E, 6F). Furthermore, upregulation of LncRNA ADAMTS9 increased the expression levels of NLRP3 in GC cells, which were abrogated by overexpressing miR-223-3p (Figure 6G, 6H).

### LncRNA ADAMTS9 triggered cisplatin-induced CR-GC cell pyroptosis by targeting miR-223-3p

**Figure 7:**
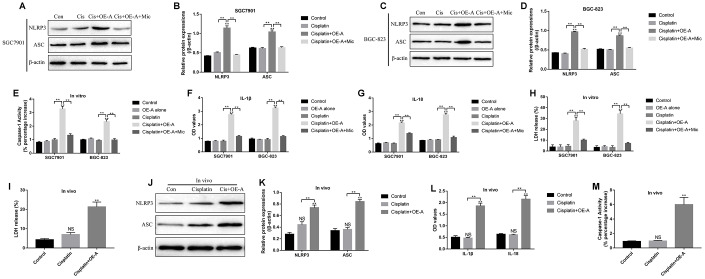
**The role of LncRNA ADAMTS9/miR-223-3p axis in the regulation of pyroptotic cell death in cisplatin treated CR-GC cells *in vitro* and *in vivo*.** Western Blot was used to detect the expression levels of NLRP3 and ASC in (**A**, **B**) CR-SGC7901 cells and (**C**, **D**) CR-BGC-823 cells. (**E**) The activity of Caspase-1 was determined in CR-GC cells. ELISA was employed to examine (**F**) IL-1β and (**G**) IL-18 expressions in the supernatants. (**H**) LDH release was measured to evaluate the formation of pores in the cell membrane. (**I**) LDH release was measured in mice cancer tissues. (**J**, **K**) The expressions of NLRP3 and ASC were determined by Western Blot in mice cancer tissues. (**L**) ELISA was used to measure IL-1β and IL-18 expressions in mice serum. (**M**) Caspase-1 activity was measured in mice cancer tissues. Each experiment repeated at least 3 times. “NS” represented “no statistical significance”, *P < 0.05, **P < 0.01.

Based on the above results, we next investigated whether LncRNA ADAMTS9/miR-223-3p/NLRP3 axis induced pyroptotic cell death in CR-GC cells treated with high-dose cisplatin. To achieve this, the CR-GC cells were pre-transfected with LncRNA ADAMTS9 overexpression vectors and miR-223-3p mimic, and subsequently treated with high-dose cisplatin for 24 h. The pyroptosis associated proteins (NLRP3, ASC, IL-18 and IL-1β) and caspase-1 activity were determined in cells and supernatants, respectively. The results showed that high-dose cisplatin alone did not affect the above proteins, while overexpression of LncRNA ADAMTS9 significantly increased the expression levels of NLRP3 and ASC in cisplatin treated CR-GC cells (Figure 7A–7D). Consistently, cisplatin or LncRNA ADAMTS9 overexpression alone had little effects on caspase-1 activity (Figure 7E), IL-1β (Figure 7F) and IL-18 (Figure 7G) expressions in CR-GC cells and their supernatants, which were promoted by cisplatin plus LncRNA ADAMTS9 overexpression treatment. Furthermore, pyroptosis was accompanied by pores formation in the membrane, which led to the release of intracellular contents, such as lactate dehydrogenase (LDH), and the results showed that LncRNA ADAMTS9 overexpression induced LDH release from CR-GC cells treated with cisplatin (Figure 7H). Of note, LncRNA ADAMTS9 triggered pyroptotic cell death in high-dose cisplatin treated CR-GC cells were abrogated by overexpressing miR-223-3p (Figure 7A–7H). The *in vitro* results were also validated by our *in vivo* experiments. Specifically, overexpression of LncRNA ADAMTS9 promoted LDH release (Figure 7I), and increased NLRP3 and ASC expression levels in cancer tissues (Figure 7J, 7K), and promoted IL-18 and IL-1β expressions in mice serum (Figure 7L). Additionally, the activity of caspase-1 was increased by LncRNA ADAMTS9 overexpression in mice tumor tissues (Figure 7M).

## DISCUSSION

Although recent study reported the involvement of LncRNA ADAMTS9-AS2 in the regulation of GC progression [24], the detailed molecular mechanisms are still not fully delineated. In this study, analysis of clinical samples showed that LncRNA ADAMTS9-AS2 was low-expressed, while miR-223-3p was high-expressed in GC tissues compared to the normal tissues, and patients with higher LncRNA ADAMTS9-AS2 and lower miR-223-3p tended to have a favorable prognosis. Further *in vitr*o gain- and loss-function experiments validated that LncRNA ADAMTS9-AS2 inhibited GC cell proliferation and mobility by sponging miR-223-3p, which were also verified in the previous study [23]. Specifically, LncRNA ADAMTS9-AS2 acted as a tumor suppressor in GC pathogenesis [23], and miR-223-3p overexpression promoted GC development [34]. Of note, LncRNA ADAMTS9-AS2 inhibited miR-223-3p expressions in lung cancer by serving as a competitive endogenous RNA [40]. Therefore, we demonstrated that LncRNA ADAMTS9-AS2 inhibited GC progression by sponging miR-223-3p.

Long-term cisplatin stimulation rendered GC patients with resistance to this chemotherapeutic drug [50, 51], which seriously limited its therapeutic efficacy in clinic. Previous literature reported that LncRNA ADAMTS9-AS2 regulated chemoresistance in clear cell renal cell carcinoma [25], glioblastoma [26] and breast cancer [27], but no publications reported the involvement of LncRNA ADAMTS9-AS2 in the regulation of cisplatin resistance in GC cells. Additionally, miR-223-3p promoted cisplatin resistance of GC cells via targeting F-box and WD repeat domain containing 7 (FBXW7) [37]. Based on this, we found that LncRNA ADAMTS9-AS2 was low-expressed, while miR-223-3p was high-expressed in CR-GC cells and ACR-GC cells, and continuous low-dose cisplatin stimulation significantly decreased LncRNA ADAMTS9-AS2, and increased miR-223-3p levels in GC cells, which suggested that the expression patterns of LncRNA ADAMTS9-AS2 and miR-223-3p were changed by cisplatin treatment. Further experiments validated that overexpressed LncRNA ADAMTS9-AS2 enhanced the inhibiting effects of high-dose cisplatin on CR-GC cell viability, which were reversed by upregulating miR-223-3p. The above results suggested that LncRNA ADAMTS9-AS2 sensitized CR-GC cells to cisplatin by targeting miR-223-3p.

Cisplatin inhibited cancer progression by triggering autophagic cell death [42], apoptosis [43], pyroptosis [17], ferroptosis [44] and necroptosis [45]. Hence we investigated the underlying mechanisms of LncRNA ADAMTS9-AS2 overexpression induced cell death in CR-GC cells treated with high-dose cisplatin. The results showed that only the inhibitors for apoptosis and pyroptosis, instead of the inhibitors for other types of cell death, rescued cell viability in cisplatin treated CR-GC cells transfected with LncRNA ADAMTS9-AS2 overexpression vectors, which suggested that LncRNA ADAMTS9-AS2 enhanced the cytotoxic effects of cisplatin on CR-GC cells by triggering apoptotic and pyroptotic cell death. Of note, previous study found that cisplatin induced lung cancer cell pyroptosis through apoptotic stimulation [17], however, future work are still needed to investigate the underlying mechanisms. In addition, we found that LncRNA ADAMTS9-AS2 activated NLRP3 inflammasome in GC cells by downregulating miR-223-3p, which were in accordance with the previous studies [47, 49]. Further results validated that LncRNA ADAMTS9-AS2 triggered cell pyroptosis in cisplatin treated CR-GC cells by regulating miR-223-3p/NLRP3 axis *in vitro* and *in vivo*.

**Figure 8:**
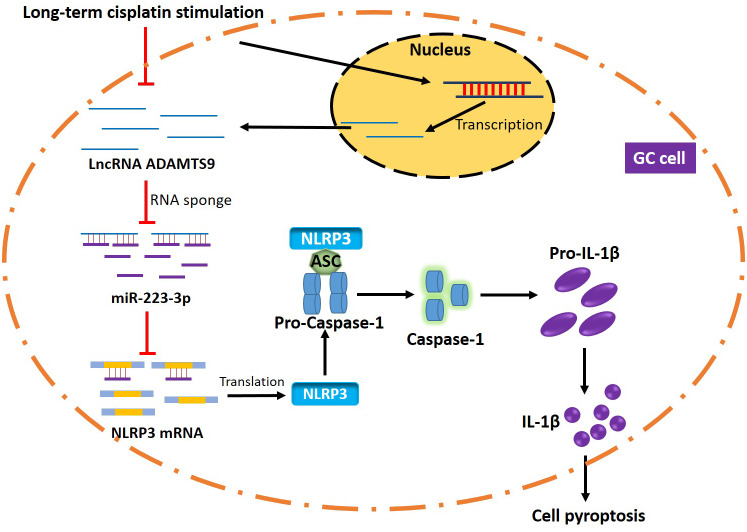
**The graphical abstract of this study.** Briefly, long-term cisplatin stimulation inhibited LncRNA ADAMTS9 and increased miR-223-3p levels in GC cells, which inhibited pyroptotic cell death by inactivating NLRP3 inflammasome. Therefore, the GC cells with aberrant gene expressions were resistant to cisplatin.

Collectively, LncRNA ADAMTS9-AS2 overexpression inhibited GC progression and sensitized CR-GC cells to cisplatin by regulating miR-223-3p/NLRP3 axis mediated cell pyroptosis (Figure 8). This study will give some insights into the molecular mechanisms of the chemoresistance generated by GC cells to cisplatin and provide new therapeutic agents for GC treatment in clinic.

## MATERIALS AND METHODS

### Clinical specimens

The GC tissues and their paired normal adjacent tissues were collected from patients (N = 45) diagnosed as GC in the First Affiliated Hospital of Harbin Medical University from 2012 to 2014. These GC patients did not receive any treatment before surgical resection. The clinicopathological features of the above patients were summarized in Table 1. All the clinical experiments were approved by the Institutional Ethics Committee of the First Affiliated Hospital of Harbin Medical University, and all the participants involved in this study signed the informed consent form.

### Cell culture

The human gastric epithelial cell line GES-1, cisplatin-sensitive GC (CS-GC) cell lines (SGC7901, MKN74, NUGC-4, HGC-27 and BGC-823) and cisplatin-resistant GC (CR-GC) cell line (SGC7901/DDP and BGC-823/DDP) were purchased from Cancer Research Institute of Beijing (China). All the cells were cultured in DMEM (Gibco, USA) containing 10% fetal bovine serum (FBS) under standard conditions (37 °C with 5% CO_2_). The GC cells were then treated with continuous low-dose cisplatin in a stepwise manner to generate acquired cisplatin-resistant GC (ACR-GC) cells in this study.

### Vectors transfection

The vectors for LncRNA ADAMTS9-AS2 overexpression and silencing were obtained from YRBIO (Changsha, China). The miR-223-3p mimic and inhibitor were designed and synthesized by Sangon Biotech (Shanghai, China). The above vectors were delivered into GC cells by using the Lipofectamine 3000 reagent purchased from Invitrogen (CA, USA) according to the manufacturer’s instruction.

### Real-time qPCR

The TRIzol reagent (Invitrogen, CA, USA) was used to extract total RNA from cancer tissues and cells, the expression levels of LncRNA ADAMTS9-AS2 and miR-223-3p were determined by using the THUNDERBIRD SYBR qPCR reagent (Toyobo, Hapan) according to the manufacturer’s protocol. The primer sequences were listed as follows: U6 (Forward: 5’-GAC TAT CAT ATG CTT ACC GT-3’, Reverse: 5’-GGG CAG GAA GAG GGC CTA T-3’), LncRNA ADAMTS9-AS2 (Forward: 5’-AGG CTG AAG TTA CAG GTC-3’, Reverse: 5’- TTG GCT CCC AGT GTC TTA-3’) and miR-223-3p (Forward: 5’-GAA GCT GTA CCT AAC ATA CCG TG-3’, Reverse: 5’- GAT TGG TCG TGG ACG TGT CG-3’).

### Western blot

The radio immunoprecipitation assay (RIPA) lysis buffer (Beyotime, Shanghai, China) was used to extract the total protein from cancer tissues and cells. Western Blot was conducted to determine the expression levels of proteins involved in this study according to the previous study [18]. The primary antibodies against NLRP3 (1:1000), β-actin (1:1000) and ASC (1:1500) were purchased from Abcam (UK). The protein bands were visualized by using the electro-chemi-luminescence (ECL) Western Blot detection kit (GE Healthcare Bio-science, USA) in keeping with the manufacturer’s instruction and quantified by Image J software.

### Cell counting kit-8 (CCK-8) assay

The cell proliferation abilities of GC cells were measured by using the commercial CCK-8 kit (AbMole, USA) according to the manufacturer’s instruction. Briefly, the cells were incubated with CCK-8 reaction solution for 4 h and the Gemini EM microplate reader (Molecular Devices, USA) was used to measure optical density (OD) values at the wavelength of 450 nm to evaluate cell proliferation.

### Cell counting assay by Trypan blue staining

The GC cells were harvested and stained with Trypan blue (Sigma, USA) according to the protocol. After that, the cells were counted under optical microscope, the cells stained with blue were regarded as dead cells. The cell viability was calculated by using the following formula: cell viability (%) = live cells/total cells * 100 %.

### Transwell assay

The GC cells were seeded into the upper floor of Transwell chambers (BD Biosciences, USA) with serum-free medium at the density of 5 × 10^4^ cells per well, and the lower chambers were added with culture medium containing 20 % fetal bovine serum (FBS). After 24 h incubation under the standard conditions, the invasive cells were fixed in methanol and stained with 0.1 % crystal violet to visualize the cells. The cells were observed and photographed under optical microscope.

### Colony formation assay

The GC cells were harvested and cultured in 6-well plates at the density of 500 cells per well for 14 days. After that, the cells were stained with crystal violet (Beyotime, China) based on the protocol provided by the manufacturer. The cells were photographed under optical microscope (ThermoFisher Scientific, USA) and the colonies containing at least 10 cells were counted.

### Flow cytometry (FCM)

The Annexin V-FITC/Propidium Iodide (PI) double-stain kit (BD Bioscience, USA) was employed to detect cell apoptosis ratio according to the manufacturer’s instruction. After that, the Flow Cytometry (FCM) (ThermoFisher Scientific, USA) was employed to measure cell apoptosis ratio.

### Enzyme linked immunosorbent assay (ELISA)

The supernatants for GC cells and mice serum were collected, the ELISA kit was used to measure the expression levels of IL-1β and IL-18 according to the protocol provided by the producer. The HRP-labeled goat anti-rabbit IgG antibodies were used as secondary antibody. The absorbance values were detected by using the microplate reader (Molecular Devices, USA) at the wavelength of 450 nm.

### Caspase-1 activity detection

The activity of caspase-1 was measured by using the Caspase-1 Activity Assay Kit (Solarbio, China) in keeping with the manufacturer’s instruction. The specimens from tissues and cells were lysed by the lysis buffer. The contents of total proteins were evaluated by Bradford method and a microplate reader was employed to examine the optical density (OD) values at the wavelength of 405 nm, which was utilized to represent caspase-1 activity according to the previous study [52].

### Lactate dehydrogenase (LDH) release assay

The LDH cytotoxicity assay kit (Beyotime, China) was purchased to measure LDH release based on the protocol provided by the manufacturer. Briefly, the GC cells were administered with different treatments, and the supernatants were harvested. The LDH reagent was used to detect LDH release at the wavelength of 490 nm according to the previous study [52].

### Dual-luciferase reporter gene system

The wild-type (Wt) and mutant (Mut) LncRNA ADAMTS9-AS2 and 3’UTR regions of NLRP3 mRNA were synthesized and cloned into the psiCHECK-2 vectors (Promega, USA). The above vectors were co-transfected with miR-223-3p mimic and miR-NC (Sangon Biotech, China) into SGC7901 and BGC-823 cells, respectively. After 48 h incubation, the dual-luciferase reporter gene system (Promega, USA) was employed to measure relative luciferase activity based on the manufacturer’s protocol.

### Pull-down assay

The biotin-labeled probes for LncRNA ADAMTS9-AS2 and 3’ UTR regions of NLRP3 mRNA were designed and synthesized by Sangon Biotech (Shanghai, China). The cells were fixed, lysed and sonicated. After centrifugation, the supernatants were used as input, and the rest part of SGC7901 and BGC-823 cells were incubated with the streptavidin Dynabeads (Invitrogen, USA) mixture containing the above probes at 30 °C overnight. After that, the lysis buffer and Proteinase K were employed to reverse crosslinking and release miR-223-3p, which were quantified by Real-Time qPCR.

### Xenograft models

The nude mice (N = 18, age 6-8 weeks) were purchased from the Experimental Animal Center of the First Affiliated Hospital of Harbin Medical University. The CR-GC cell line (SGC7901/DDP) were subcutaneously injected into the back flank of each mouse, the LncRNA ADAMTS9-AS2 overexpression vectors were also injected into the tumor formation sites. The above mice were equally divided into 3 groups, including control group, cisplatin alone group and cisplatin plus overexpressed LncRNA ADAMTS9-AS2 group, respectively. Until tumor volume reached 1 mm^3^, the high-dose cisplatin (10 μg/ml) were administered for 2 weeks, the cancer tissues and serum were collected from the mice for further investigation. All the animal experiments were approved by the Animal Management Center of the First Affiliated Hospital of Harbin Medical University.

### Statistical analysis

All the data involved in this study were collected and represented as Mean ± Standard Deviation (SD). The SPSS 18.0 software was used to analyze the data. Specifically, student’s t-test method was used to compare the differences between two groups. The one-way Analysis of Variance (ANOVA) method was employed to compare the differences among multiple groups. The Pearson correlation analysis was used to analyze the correlation of LncRNA ADAMTS9-AS2 and miR-223-3p in GC tissues. The prognosis for GC patients was analyzed by Kaplan-Meier survival analysis. Each experiment in this study repeated at least 3 times. The analysis results were visualized by using the Graphpad Prism 5 software. “NS” indicated “No statistical significance”, *P < 0.05 and **P < 0.01.

## Supplementary Material

Supplementary Figure 1
