# Supplementary material for: LncRNA ADAMTS9-AS2 inhibits gastric cancer (GC) development and sensitizes chemoresistant GC cells to cisplatin by regulating miR-223-3p/NLRP3 axis
Source: Aging (Albany NY). 2020 Jun 9;12(11):11025–41. doi: 10.18632/aging.103314 (PMC7346038; doi:10.18632/aging.103314)
Supplement: Supplementary Figure 1 [file aging-12-103314-s001..pdf]

SUPPLEMENTARY FIGURE

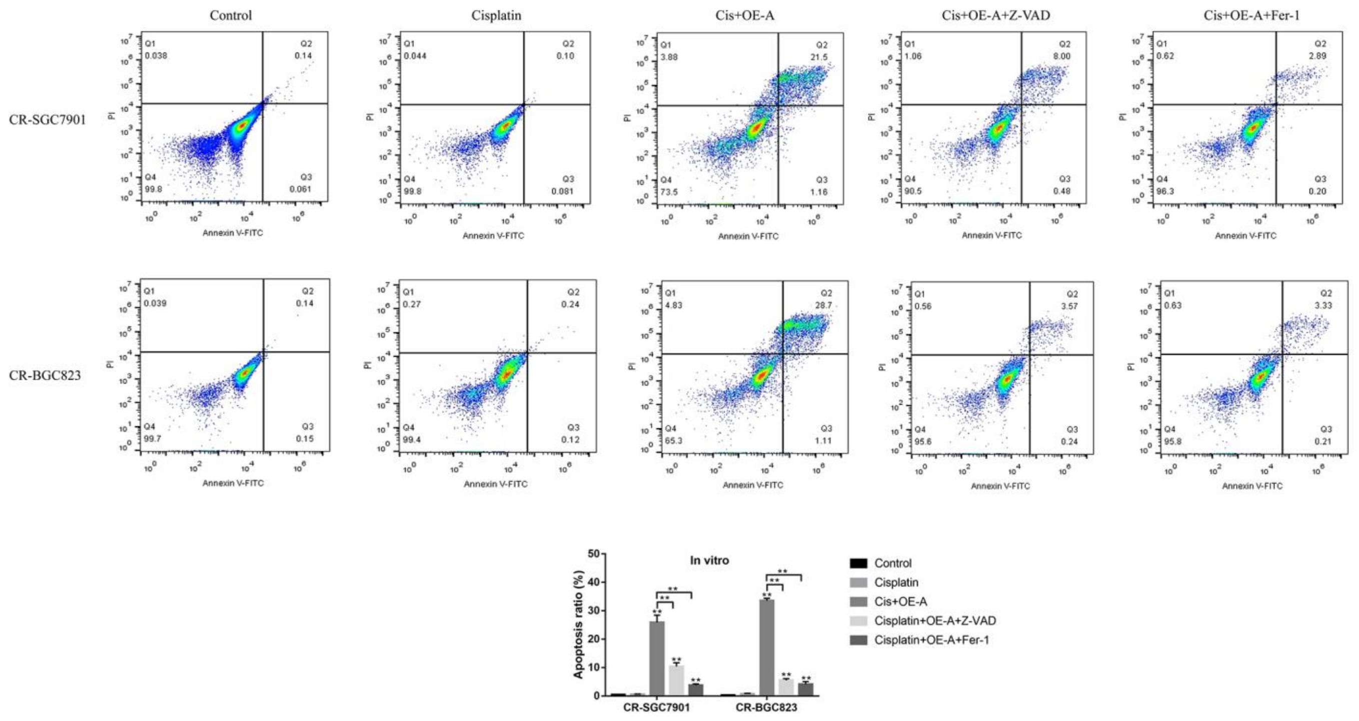

Supplementary Figure 1. FCM assay was performed to detect cell apoptosis ratio. The cells were stained with PI and Annexin V-FITC, and cell apoptosis was measured by FCM. ***P* < 0.01.
